# Transepithelial electrical resistance (TEER): a functional parameter to monitor the quality of oviduct epithelial cells cultured on filter supports

**DOI:** 10.1007/s00418-015-1351-1

**Published:** 2015-07-31

**Authors:** Shuai Chen, Ralf Einspanier, Jennifer Schoen

**Affiliations:** College of Life Science, Hebei University, Baoding, 071002 Hebei People’s Republic of China; Institute of Reproductive Biology, Leibniz Institute for Farm Animal Biology, Wilhelm-Stahl-Allee 2, 18196 Dummerstorf, Germany; Institute of Veterinary Biochemistry, Department of Veterinary Medicine, Freie Universität Berlin, Oertzenweg 19b, 14163 Berlin, Germany

**Keywords:** TEER, Oviduct epithelial cells, Culture quality, Tight junctions

## Abstract

Cultivation of oviduct epithelial cells on porous filters fosters in vivo-like morphology and functionality. However, due to the optical properties of the filter materials and the cells’ columnar shape, cell quality is hard to assess via light microscopy. In this study, we aim to evaluate transepithelial electrical resistance (TEER) measurement as a prognostic quality indicator for the cultivation of porcine oviduct epithelial cells (POEC). POEC were maintained in four different types of media for 3 and 6 w to achieve diverse culture qualities, and TEER was measured before processing samples for histology. Culture quality was scored using morphological criteria (presence of cilia, confluence and cell polarity). We furthermore analyzed the correlation between cellular height (as a measure of apical–basal polarization) and TEER in fully differentiated routine cultures (biological variation) and in cultures with altered cellular height due to hormonal stimulation. Fully differentiated cultures possessed a moderate TEER between 500 and 1100 Ω*cm^2^. Only 5 % of cultures which exhibited TEER values in this defined range had poor quality. Sub-differentiated cultures showed either very low or excessively high TEER. We unveiled a highly significant (*P* < 0.0001) negative linear correlation between TEER and epithelial height in well-differentiated cultures (both routine and hormone stimulated group). This may point toward the interaction between tight junction assembly and epithelial apical–basal polarization. In conclusion, TEER is a straightforward quality indicator which could be routinely used to monitor the differentiation status of oviduct epithelial cells in vitro.

**Electronic supplementary material:**

The online version of this article (doi:10.1007/s00418-015-1351-1) contains supplementary material, which is available to authorized users.

## Introduction

Culturing epithelial cells on porous filter supports mimics in vivo-like conditions by allowing nutrient supply from the basolateral compartment and therefore promotes cell differentiation and closer (morphological as well as functional) similarity to the native tissue (Zegers et al. 2003). Transepithelial electrical resistance (TEER) measurement is used to assess the barrier function of epithelial cells on these porous supports. When assessing the electrical impedance, a continuous current passes through the cells on both transcellular and paracellular paths (Powell 1981). The transcellular resistance is primarily made up by the apical and basolateral plasma membrane, while the paracellular resistance is a result of cell–substrate as well as cell–cell contacts. It is known that specific tight junction proteins largely influence epithelial resistance (Anderson 2001; Lo et al. 1999). Hence, the TEER value reflects physical structures and properties of filter-grown epithelial cultures. So far, TEER measurement has been mainly applied for assessing the permeability of tight junctions or the membrane perturbation by toxicants on intestinal and kidney epithelial cell lines (Narai et al. 1997; Velarde et al. 1999; Madgula et al. 2007).

The oviduct epithelium is the reproductive venue where transport and maturation of gametes, fertilization and cleavage-stage embryonic development occur (Coy et al. 2012). Taking advantage of porous filter supports, recently primary oviduct epithelial cultures with improved cell differentiation have been generated from species including: monkey, human, pig and cattle (Chen et al. 2013b; Levanon et al. 2010; Palma-Vera et al. 2014; Rajagopal et al. 2006; Gualtieri et al. 2012). Tools allowing non-invasive quality control are highly desirable for these differentiated models as filter-supported techniques are more complex compared to basic cell cultures. Moreover, these models are normally maintained over prolonged time periods (up to 6 w), which further necessitates quality monitoring. There are two major factors interfering with routine morphological quality control via light microscopy: (1) The commercial membrane materials are polycarbonate (PC) or polyester (PET) with a thickness ≥10 µm; (2) cells differentiate in the apical–basal axis reaching a height up to 18 µm (Chen et al. 2013b). The polarization status in the apical–basal dimension (columnar shape) is therefore hard to assess by microscopy of living cells, although it is a crucial quality criterion in this cell type.

Recently, we validated a standardized culture model of porcine oviduct epithelial cells (POEC) in large-scale trials, which exhibits distinct hormonal responsiveness during estrous cycle simulation (Chen et al. 2013a, b). In this follow-up study, we evaluate whether TEER measurement could serve as a straightforward quality indicator for differentiated oviduct epithelial models. To obtain cultures with diverse qualities, cells were maintained in four different types of media for 3 and 6 w. We examined the association between TEER values and the morphological features of cultured cells, and further classified TEER values into ranges for quality indication. We also unveiled how TEER values correlate with apical–basal polarization (measured as cellular height) in routinely cultured as well as hormonally stimulated cells.

## Materials and methods

### Materials

If not otherwise indicated, all cell culture reagents and materials (including 24-well PET Millicell inserts PIHT12R48 and fetal bovine serum FBS S0115) were acquired from Merck Millipore. Chemicals for histological procedures were obtained from Carl Roth. EVOM2 Epithelial Voltohmmeter together with STX2 electrode was purchased from World Precision Instruments (WPI).

### Tissue samples and cell culture

Oviducts were collected from 6-month-old gilts (Large White × German Landrace) in the local slaughterhouse immediately after slaughter. Cell isolation and filter-supported cultivation were performed following the protocol previously reported by our group (Miessen et al. 2011; Chen et al. 2013b). Cell cultures with divergent qualities were generated by using four types of media: *(M1)* Ham’s F12 with 10 % charcoal-stripped FBS (S181F, Biowest); *(M2)* two parts of M1 plus one part of the correspondent 3T3-conditioned medium; *(M3)* Ham’s F12 with 10 % FBS (S0115, Biochrom); and *(M4)* two parts of M3 plus one part of the corresponding 3T3-conditioned medium. All media were supplemented with 100 U/mL penicillin, 100 µg/mL streptomycin, 50 µg/mL gentamicin, 1 µg/mL amphotericin B, 10 mg/mL reduced glutathione and 10 mg/mL ascorbic acid (Sigma). 3T3-conditioned medium was prepared following the standard protocol (Freshney 2005). Air/liquid culture was achieved by removing the medium (200 µl) in the apical compartment 48 h after seeding.

### Morphology and morphometric assessment

To harvest cells, membranes were cut out from inserts and washed in PBS followed by 2 h fixation in Bouin’s solution. Subsequently, membranes were cut in half, vertically settled in 2 % agarose and post-fixed in 4 % formalin. After dehydration in ascending ethanol series, samples were embedded in paraplast. Approximately, 3-µm-thick sections were prepared from the middle part of the membrane. Finally, slides were stained with hematoxylin/eosin (HE) and analyzed by light microscopy.

Cultures grown in different media were scored using qualitative morphological criteria including: presence of cilia, cell polarity (columnar shape) and confluence of the culture (Table 1). Scores for “cilia” and “polarity” were assigned ranging from 0 to 3, while scores for “confluence” were assigned ranging from 0 to 2. POEC were cultured for 3w (*N* = 4 animals) and 6w (*N* = 6 animals) in all four types of media, respectively. Detailed scores for each criterion and relevant TEER values are listed in the supplementary table (S1).

**Table 1:** Evaluation criteria for morphological scoring of POEC

Score	Cilia	Polarity	Confluence
3	Dense cilia	Cellular height ≥ 10 µm	/
2	Cilia in certain areas	5 μm < cellular height < 10 μm	Fully confluent
1	Very few cells with cilia	2 μm < cellular height < 5 μm	Partly confluent
0	No cilia	No cellular polarity visible	No or few cells on membrane

To assess the correlation between TEER and apical–basal polarization of the cell culture, we further selected cellular height as a quantitative morphometric parameter. As all cultures in M4 achieved highest quality, M4 was used for this part of the study. Histomorphometric height measurements were taken in (1) routine cell cultures: cells grown for 3w (*N* = 23 animals) and 6w (*N* = 23 animals); 2) hormonal stimulation experiment: cells treated with 35 ng/ml progesterone (P4; Sigma-Aldrich) and 10 pg/mL 17β-estradiol (E2; Sigma-Aldrich) for the last 10 days of the 3w (*N* = 11 animals) and 6w (*N* = 11 animals) culture period. As we reported earlier, this stimulation protocol changes the functional characteristics of the oviduct epithelial cells and allows simulation of the changes observed during the estrous cycle in vivo (Chen et al. 2013a). Controls (cells treated with ethanol solvent only) were included in the hormonal stimulation experiment. A total of 15 histomorphometric height measurements were taken for each sample following a recently published protocol (Chen et al. 2013a).

### TEER measurement

We routinely performed TEER measurements for all samples shortly before fixation. To eliminate the influence of temperature, measures were performed within 5 min after taking the culture plates out of the incubator. Within this time, samples did not show any reading drift. Before measurement, electrodes were equilibrated and sterilized according to the manufacturer’s recommendations. Two hundred microliters of culture medium was added in the upper compartment of the cell culture system. The ohmic resistance of a blank (culture insert without cells) was measured in parallel. To obtain the sample resistance, the blank value was subtracted from the total resistance of the sample. The final unit area resistance (Ω*cm^2^) was calculated by multiplying the sample resistance by the effective area of the membrane (0.33 cm^2^ for 24-well Millicell inserts).

### Statistics

Statistical data were analyzed by SPSS Statistics 20 for Windows. Correlation coefficient was assessed by Pearson test, while linear regression analyses were used to determine the slope between TEER values and cellular heights. A *P* value < 0.01 was considered statistically significant.

## Results

### TEER and morphological quality of POEC

**Fig. 1:**
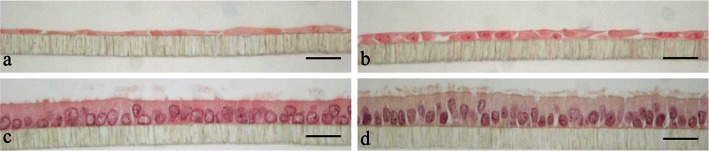
HE staining of primary porcine oviduct epithelial cells (POEC) cultured in media M1 (**a**), M2 (**b**), M3 (**c**) and M4 (**d**), respectively, for 6w. M1: Ham’s F12 + 10 % charcoal-stripped FBS; M2: M1 enriched with corresponding 3T3-conditioned medium at a ratio of 2:1(V: V); M3: Ham’s F12 + 10 % FBS; M4: M3 enriched with corresponding 3T3-conditioned medium at a ratio of 2:1(V: V); magnification ×400,* scale bars* 20 μm

**Fig. 2:**
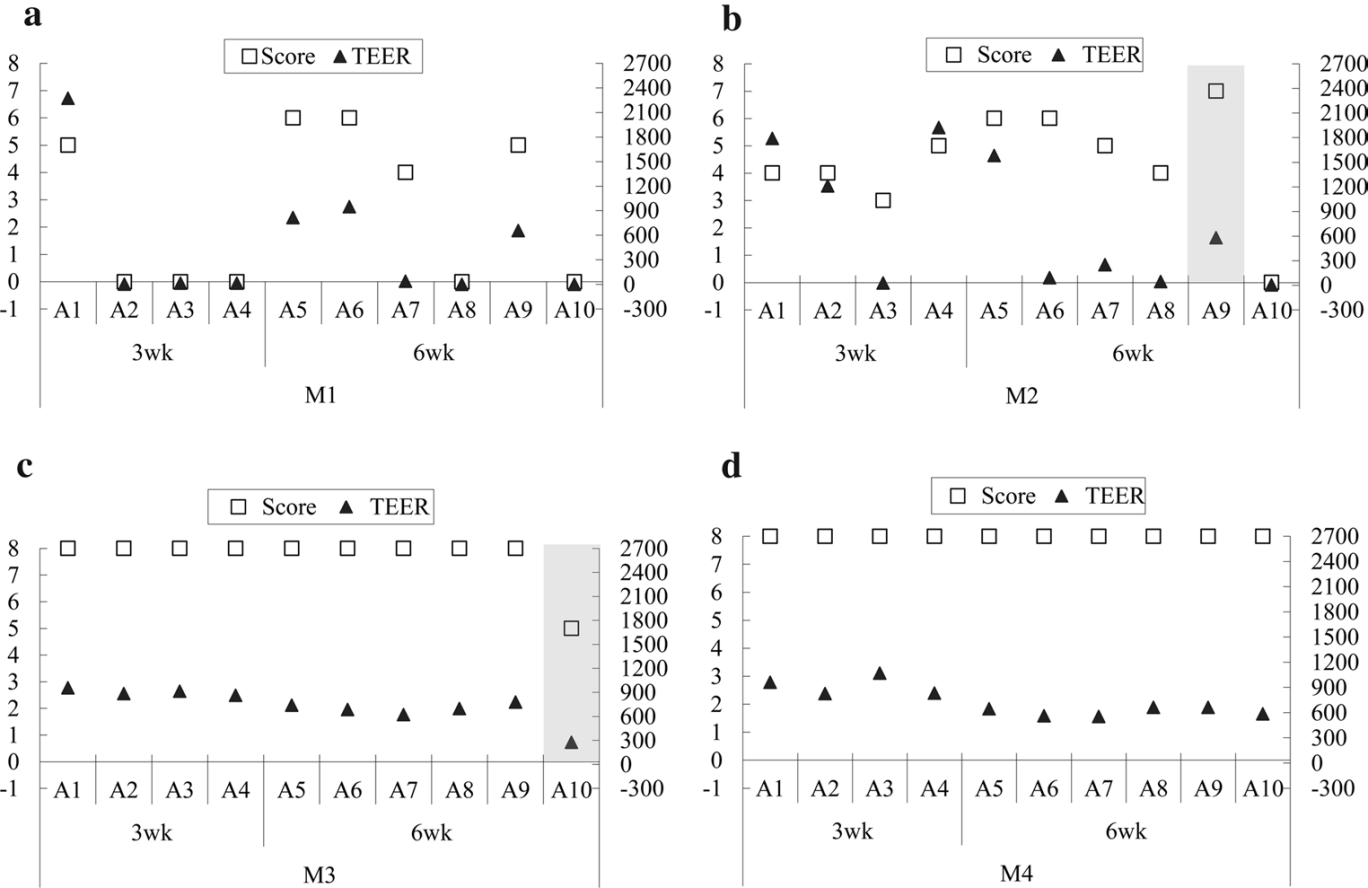
Morphological scores and corresponding TEER values of POEC after 3w (*N* = 4 animals) and 6w (*N* = 6 animals) cultivation. Cells are grown in media M1 (**a**), M2 (**b**), M3 (**c**) and M4 (**d**), respectively. Left *y* axis: morphological scores; right *y* axis: TEER values (Ω*cm^2^). *Shadow area* in (**b**) highlights a well-differentiated sample with moderate TEER value; *shadow area* in (**c**) highlights a poorly differentiated sample with low TEER value

Cells exhibited disparate architectures when subjected to different media. Overall, both charcoal-stripped media lead to poor morphological quality: Cells were dedifferentiated, flat or dwarf, lost cilia and secretory protrusions (Fig. 1a, b), with an average morphology score of 2.6 in M1 and 4.2 in M2 (Fig. 2a, b). Their correspondent TEER values varied tremendously from 0 to 2270 Ω*cm^2^. In contrast, 9 out of 10 cultures in M3, and all cultures in M4 achieved highest quality (score = 8). Cells were highly polarized, confluent and retained a mix of ciliated and secretory cells (Fig. 1c, d). They displayed consistent and moderate TEER readings from 729 to 1070 Ω*cm^2^ (Fig. 2c, d). The best culture (score = 7) in M2 also showed a medium TEER output (Fig. 2b); the only outlier in M3, which had a low morphology score of 5 (confluence was given, see S1), exhibited a low TEER reading (Fig. 2c).

**Fig. 3:**
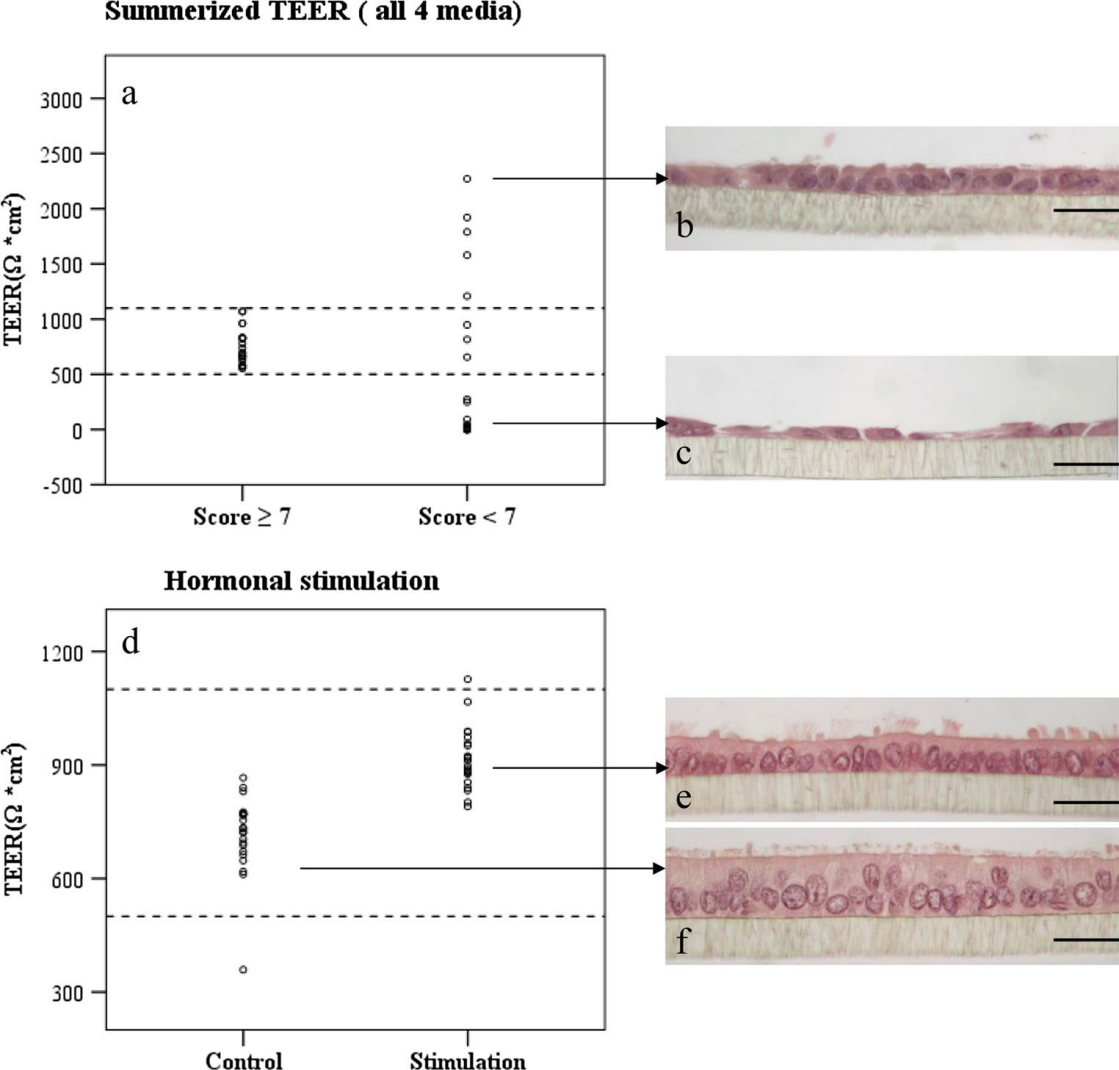
TEER in POEC cultures with divergent quality and with or without hormonal stimulation. **a** TEER of well-differentiated (score ≥ 7) and non-differentiated (score < 7) POEC cultures. **d** TEER of POEC cultures in the hormonal stimulation experiment. Treated group: Cells are first cultured for 11 or 32 days and then treated with 35 ng/ml P4 and 10 pg/ml E2 for 10 days; control group: cells received solvent only. **b** Representative histological picture of a POEC culture with high TEER (1579 Ω*cm^2^). **c** POEC culture with low TEER (25.25 Ω*cm^2^). **e** POEC culture under hormonal stimulation (P4 dominance) with a TEER of 840 Ω*cm^2^. **f** Control POEC in the hormonal stimulation experiment (solvent only; TEER of 724 Ω*cm^2^). (**b**, **c**, **e**, **f**) HE staining, magnification ×400,* scale bars* 20 µm

To better visualize the relation between TEER and culture quality, we pooled data from above four media and divided them into two groups: good quality (score ≥ 7) and poor quality group (score < 7). TEER of all morphological intact cultures fell into a tight range from 500 to 1100 Ω*cm^2^, while 85 % of cultures with low morphological quality possessed resistance either exceeding 1100 Ω*cm^2^ or below 500 Ω*cm^2^ (Fig. 3a). Histological investigation further revealed that partway differentiated epithelial layers tend to yield over high TEER output (Fig. 3b). Contrarily, completely undifferentiated cultures (no visible polarization) had extremely low TEER values (Fig. 3c). Similarly, in the hormonal stimulation experiment >95 % of the cultures exhibited the proposed TEER range. All of them demonstrated fully differentiated morphology (Fig. 3d). The hormonally stimulated group (P4 domination) tends to possess higher TEER outputs but lower cellular height (Fig. 3e, f).

### TEER negatively correlates with cellular height

**Fig. 4:**
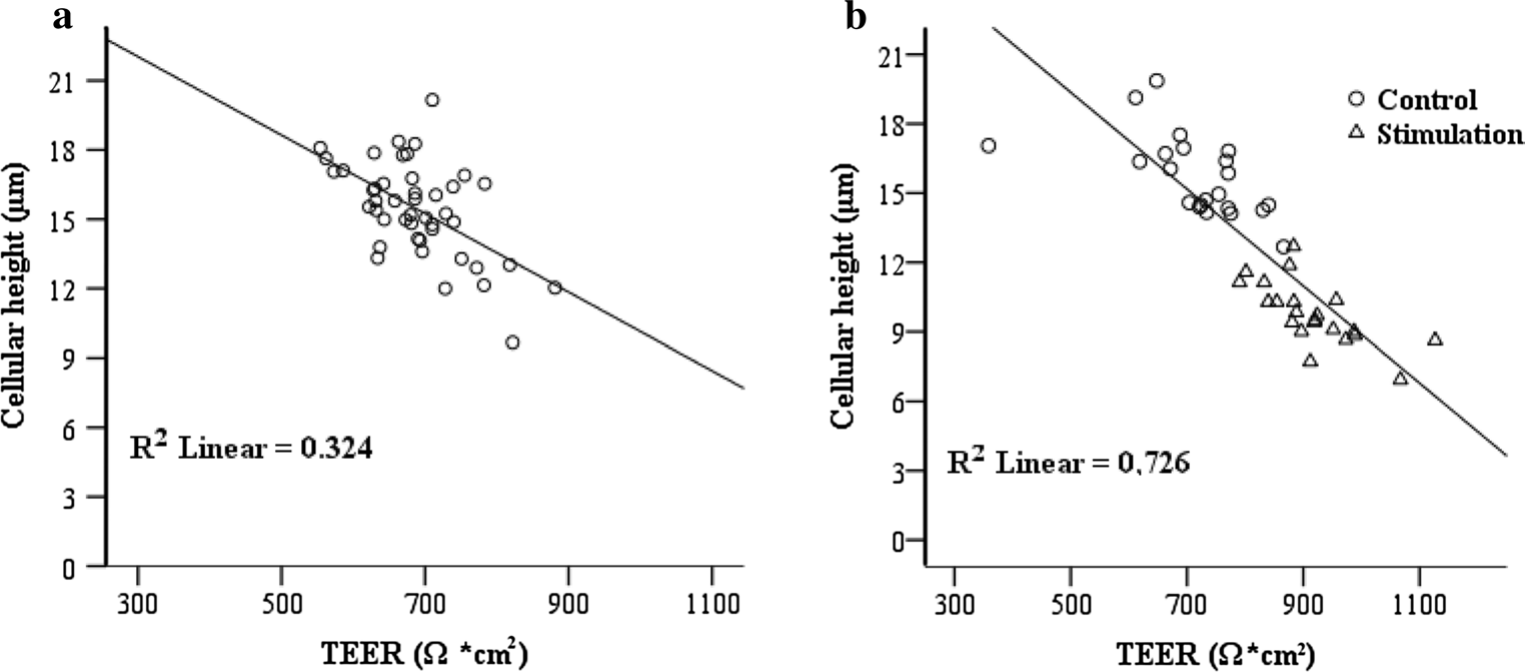
Negative correlation between TEER and cellular height. **a** POEC grown in M4 for 3w (*N* = 23 animals) and 6w (*N* = 23 animals; *n* total samples = 46; *R* = −0.569, *P* < 0.0001). **b** Hormonal stimulation experiment (*n* total samples = 44). Cells (*N* = 11 animals) are subjected to hormonal or solvent treatment during either 3w or 6w of culture in M4 (*R* = −0.852, *P* < 0.00001)

As all cultures tested in M4 were well differentiated, we selected medium M4 for further analysis. When we expanded experimental trials up to 46 in total (*N* = 23 animals, cultured for 3w and 6w, respectively), all TEER values turned out to fit in our proposed TEER range (Fig. 4a). Furthermore, a significant (*R* = −0.569, *P* < 0.0001) negative linear correlation was observed between TEER and cellular height, with the regression equation of *Y* = 27.132–0.017X.

We also analyzed the correlation between these two parameters in the hormonal stimulation experiment. Again, TEER values revealed similar negative correlation with cellular height (*R* = −0.852, *P* < 0.00001, Fig. 4b) with a linear regression equation of *Y* = 29.840–0.021X.

## Discussion

The recent advance of filter-supported oviductal epithelial cultures, which proved closer tissue-like morphology and biology than conventional cultures, necessitates a suitable monitoring technique to assess their culture qualities. After systematic analysis of TEER measurement in conjunction with morphological features of POEC achieved in varied types of media, we clearly revealed how the electrical impedance associates with differentiation status of epithelial cultures. Furthermore, we may conclude a defined TEER range for high-quality cultures (500–1100 Ω*cm^2^ in 24-well PET inserts, 0.4 µm pore size), which could be used as guidance for the cultivation of oviduct epithelial cells on filter supports. In the study presented here, 90 % of cultures with deprived morphology had TEER values below 500 or above 1100 Ω*cm^2^. In contrary, only 5 % of cultures which exhibited TEER values between 500 and 1100 Ω*cm^2^ had poor quality.

When considering the epithelial layer as a simple electrical structure, it could be described as two parallel units: a transcellular unit and a paracellular unit which is influenced mainly by tight junctions, as well as intercellular spaces and cell-substrate contacts (Lo et al. 1999; Powell 1981). According to the permeability of epithelial tissues, they could be classified into two categories: leaky and tight epithelia. Epithelia are considered leaky, when their TEER is below 1000 Ω*cm^2^, with a paracellular conductance greater than transcellular conductance (Powell 1981). Oviduct epithelium is described to be leaky (Leese 1988); therefore, in our POEC cultures, the TEER measurement largely reflected the changes in paracellular unit, mainly the elaboration of strands of tight junctions (Cereijido et al. 2008; Anderson 2001; Claude 1978; Claude and Goodenough 1973).

It is widely assumed that low TEER indicates low quality of cultured epithelial cells; however, here we clearly demonstrate that excessive transepithelial resistance in cells originating from a leaky epithelium is associated with a sub-differentiated culture status.

The proposed TEER range to forecast the quality of oviductal epithelia in vitro is further validated by comparison with well-differentiated cultures from other species: The primary culture of monkey oviduct epithelium, which closely resembled native tissue, exhibited a TEER of 750 Ω*cm^2^ by day 20 of culture (Rajagopal et al. 2006). When cultured for 17d, oviductal epithelial cells from rabbits at estrous showed a TEER of 851 Ω*cm^2^ (Edwards and Leese 1993). As in our experiment, both studies employed the Ag/AgCl electrode method for their resistance measurement. Investigations applying Ussing chambers produced relatively lower TEER values between 150 and 234 Ω*cm^2^ (Mahmood et al. 2001; Rajagopal et al. 2006). A study comparing both methods on the same cell type and culture system found around threefold lower TEER values when using the Ussing chamber (Rajagopal et al. 2006). This may be explained due to the different geometries of the two devices, leading to discrepancies in the current path/path lengths cross through the membrane, hence yielding divergent resistance outputs.

Apart from the inherent characteristics of epithelial cells, other factors also affect the transepithelial impedance. It has been shown that material and physical properties of the membrane support could affect cell morphology and cell-substrate contacts, therefore leading to divergent TEER values (Lo et al. 1999; Thwaites et al. 1993). The results we generated in this study are all based on PET (0.4 µm pore size) membranes. Besides, previous studies indicated that temperature has strong influence on the TEER measurement and may skew results (Matter and Balda 2003).

Finally, taking advantage of our standardized POEC model, we were able to quantify the cell polarization along the apical–basal axis and link it to electrical impedance assessment using TEER. To our knowledge, we could demonstrate for the first time a fine-tuned inversed correlation between cellular height, a parameter reflecting the degree of apical–basal polarity and TEER which mainly corresponds to the strength of cellular junctions.

It has been reported that in epithelial tissues, many tight junction complexes (e.g., CRB3, Par) are involved in the polarization process (Shin et al. 2006; Etienne-Manneville and Hall 2003). However, the underlying molecular mechanisms which co-regulate these two events need to be clarified in further studies. The mathematical equation we provided may facilitate the prediction of porcine oviductal epithelial cell height based on a known TEER value.

To conclude, TEER measurement is a non-invasive tool for the quality assessment of filter-based oviduct epithelial cultures. We hereby presented a proper TEER range which could be used to predict the apical–basal polarization in these cultures. Furthermore, we firstly unveiled the inverse relationship between epithelial height and impedance, which might promote our understanding of the interconnection between apical and basal polarity and tight junction assembly.

## Electronic supplementary material

Supplementary material 1 (DOCX 18 kb)
