# Supplementary material for: Transepithelial electrical resistance (TEER): a functional parameter to monitor the quality of oviduct epithelial cells cultured on filter supports
Source: Histochem Cell Biol. 2015 Jul 31;144(5):509–15. doi: 10.1007/s00418-015-1351-1 (PMC4628619; doi:10.1007/s00418-015-1351-1)
Supplement: Supplementary file 1 — Supplementary material 1 (DOCX 18 kb) [file 418_2015_1351_MOESM1_ESM.docx]

**S1 Detailed morphological scores and corresponding TEER values**

|  |  | **3w** | | | |  | **6w** | | | | | |
| --- | --- | --- | --- | --- | --- | --- | --- | --- | --- | --- | --- | --- |
|  | **Criteria** | **A1** | **A2** | **A3** | **A4** |  | **A5** | **A6** | **A7** | **A8** | **A9** | **A10** |
| **M1** | **Cilia** | 1 | 0 | 0 | 0 |  | 2 | 2 | 1 | 0 | 2 | 0 |
|  | **Polarity** | 2 | 0 | 0 | 0 |  | 2 | 2 | 1 | 0 | 1 | 0 |
|  | **Confluence** | 2 | 0 | 0 | 0 |  | 2 | 2 | 2 | 0 | 2 | 0 |
|  | **TEER** | 2270 | 2 | 15 | 15 |  | 815 | 947 | 37 | 0 | 656 | 0 |
| **M2** | **Cilia** | 0 | 1 | 0 | 1 |  | 2 | 2 | 1 | 1 | 3 | 0 |
|  | **Polarity** | 2 | 1 | 2 | 2 |  | 2 | 2 | 2 | 1 | 2 | 0 |
|  | **Confluence** | 2 | 2 | 1 | 2 |  | 2 | 2 | 2 | 2 | 2 | 0 |
|  | **TEER** | 1789 | 1209 | 25 | 1921 |  | 1579 | 91 | 249 | 44 | 576 | 0 |
| **M3** | **Cilia** | 3 | 3 | 3 | 3 |  | 3 | 3 | 3 | 3 | 3 | 2 |
|  | **Polarity** | 3 | 3 | 3 | 3 |  | 3 | 3 | 3 | 3 | 3 | 1 |
|  | **Confluence** | 2 | 2 | 2 | 2 |  | 2 | 2 | 2 | 2 | 2 | 2 |
|  | **TEER** | 955 | 883 | 912 | 863 |  | 957 | 818 | 847 | 841 | 917 | 56 |
| **M4** | **Cilia** | 3 | 3 | 3 | 3 |  | 3 | 3 | 3 | 3 | 3 | 3 |
|  | **Polarity** | 3 | 3 | 3 | 3 |  | 3 | 3 | 3 | 3 | 3 | 3 |
|  | **Confluence** | 2 | 2 | 2 | 2 |  | 2 | 2 | 2 | 2 | 2 | 2 |
|  | TEER | 961 | 826 | 1070 | 832 |  | 799 | 733 | 729 | 865 | 802 | 908 |
